# Dose-finding designs using a novel quasi-continuous endpoint for multiple toxicities

**DOI:** 10.1002/sim.5737

**Published:** 2013-01-21

**Authors:** Monia Ezzalfani, Sarah Zohar, Rui Qin, Sumithra J Mandrekar, Marie-Cécile Le Deley

**Affiliations:** a Biostatistics Department, Institut Gustave-RoussyVillejuif, France; b INSERM, U717, Biostatistics DepartmentParis, France; c INSERM, U872, Centre de Recherche des Cordeliers, Université Paris 5, Université Paris 6Paris, France; d Mayo ClinicRochester, MN, U.S.A.; e Université Paris-Sud 11Paris, France

**Keywords:** phase I, dose-finding design, continual reassessment method, quasi-continuous endpoint, multiple toxicity score, oncology, molecularly targeted agents, isotonic regression

## Abstract

The aim of a phase I oncology trial is to identify a dose with an acceptable safety profile. Most phase I designs use the dose-limiting toxicity, a binary endpoint, to assess the unacceptable level of toxicity. The dose-limiting toxicity might be incomplete for investigating molecularly targeted therapies as much useful toxicity information is discarded. In this work, we propose a quasi-continuous toxicity score, the total toxicity profile (TTP), to measure quantitatively and comprehensively the overall severity of multiple toxicities. We define the TTP as the Euclidean norm of the weights of toxicities experienced by a patient, where the weights reflect the relative clinical importance of each grade and toxicity type. We propose a dose-finding design, the quasi-likelihood continual reassessment method (CRM), incorporating the TTP score into the CRM, with a logistic model for the dose–toxicity relationship in a frequentist framework. Using simulations, we compared our design with three existing designs for quasi-continuous toxicity score (the Bayesian quasi-CRM with an empiric model and two nonparametric designs), all using the TTP score, under eight different scenarios. All designs using the TTP score to identify the recommended dose had good performance characteristics for most scenarios, with good overdosing control. For a sample size of 36, the percentage of correct selection for the quasi-likelihood CRM ranged from 80% to 90%, with similar results for the quasi-CRM design. These designs with TTP score present an appealing alternative to the conventional dose-finding designs, especially in the context of molecularly targeted agents.

## 1. Introduction

Phase I oncology trials aim to evaluate the safety of a new agent to identify a dose to be recommended (RD) for further evaluation. Toxicity remains the primary endpoint of such trials. Although toxicity is intrinsically multidimensional with different body systems possibly involved and multiple toxicity events may be observed in a single patient, conventional phase I trial designs are usually based on a binary endpoint of dose-limiting toxicity (DLT). The common practice of reducing the toxicity assessment to a binary indicator is perhaps an oversimplification of the multidimensional clinical reality: (i) the relative severity of different DLTs is ignored; (ii) the moderate toxicity events below the threshold of DLT is ignored; and (iii) the multiplicity of the events is neglected as only the worst grade is used to define the DLT. The landscape of oncology drug development has recently changed with the emergence of targeted agents available for testing. These new drugs appear more likely to induce multiple moderate toxicity rather than DLTs 1–2. In this context, the use of DLT criteria to lead the dose escalation and determine the RD deserves re-examination.

Different toxicity scoring systems have been proposed to measure quantitatively and comprehensively the overall severity of multiple toxicities for a patient, using an equivalent toxicity score (ETS) 3 or including total toxicity burden (TTB) 4–5. ETS is defined as the sum of the greater observed adjusted grade (minus 1) and a decimal value representing the accumulation of other types of toxicities defined using a logistic function. TTB is defined as the sum of the weights of all toxicities experienced by a patient, where weight reflects the relative clinical importance of each grade and type of toxicity 4. As the same arithmetic sum of toxicity can correspond to clinical situations of different toxicity burdens, we have proposed another flexible toxicity endpoint, thereafter called total toxicity profile (TTP), which is computed as the Euclidean norm of the weights rather than the arithmetic sum. Similar to the ETS and TTB, the TTP results in a quasi-continuous toxicity measure.

Current dose-finding designs that use the binary DLT for dose escalation and determination of RD, including continual reassessment method (CRM), are to be revamped to accommodate quasi-continuous toxicity endpoint. The novelty lies in the construction of an appropriate dose–toxicity function and solicitation of a target toxicity score. Several authors have proposed dose-finding methods applicable for a toxicity score 4–3. Yuan *et al*. suggested incorporating a numeric toxicity score, as a fractional event after normalization, into the CRM using the quasi-Bernoulli likelihood. They developed a new method, the quasi-CRM (QCRM), considering an empiric modeling of the dose–toxicity relationship, in a Bayesian framework 6. When the binary DLT outcome is used, several variants of the original CRM have been proposed, including different choices for the underlying parametric model for the dose–toxicity relationship and different frameworks for the estimation of parameters. The two most commonly used models in the CRM literature are the empiric (power) function and the one-parameter logistic function, as proposed by OʼQuigley *et al*. in 1991 8. The logistic regression model is widely used in epidemiology and biostatistics, and in particular in bioassays 9, because of its ability to model a wide class of sigmoid curves that present a good fit for many dose–response relationships. Although initially developed in a Bayesian framework, it has been shown that the CRM can easily be used in a more classical framework (likelihood theory) 10–11. The frequentist inference avoids the solicitation of a prior distribution for the parameter to be estimated and may thus be more easily understood and accepted by the clinicians. The investigators are usually more comfortable by starting the trial with a ‘ 3 + 3’ design 12 before switching to a model-based approach once a toxicity is observed.

The objective of the present work, motivated by the different concerns listed earlier, is to propose a novel phase I dose-finding design, the quasi-likelihood CRM (QLCRM), incorporating the TTP score into the CRM, in a frequentist framework. This method is based on a logistic modeling of the dose–toxicity relationship, both for the dose escalation process and for the identification of the RD at the end of the trial. In this paper, we compared our design with three other designs allowing for a quasi-continuous toxicity endpoint: the model-based QCRM proposed by Yuan *et al*. 6 and two nonparametric designs, the unified approach (UA) published by Ivanova and Kim 7 and the extended isotonic design (EID) proposed by Chen *et al*. 3. We evaluated the dose escalation process and the identification of the RD of each of these methods, using the same endpoint, that is, the TTP, across the different methods.

In Section 2, we first define the toxicity score TTP and then describe the QLCRM method implemented for both the dose escalation process and the identification of the RD at the end of the trial. This section ends up with a short description of the alternative dose-finding methods that we evaluate 6,3. In Section 3, we detail the simulations that we used to compare these different methods. Section 4 presents the results of this comparison, in terms of percentage of dose recommendation, allocated dose percentage, distribution of the toxicity scores, and number of observed DLTs during the trial, as well as the convergence of the percentage of correct selection (PCS), under various dose–toxicity scenarios. We conclude with our discussion in Section 5.

## 2. Methods

Let *D* be the sample space of prespecified discrete doses explored in the trial *D* = {*d*_1_, … ,*d*_*k*_, … ,*d*_*K*_}with *d*_1_ < *d*_2_ < … < *d*_*k*_ < … < *d*_*K*_. We sequentially accrue the patients by cohorts.

### 2.1. Total toxicity profile and acceptable toxicity target

The TTP is a quasi-continuous toxicity measure combining the multiple toxicity grades according to their relative clinical importance, using their respective prespecified weights.

#### 2.1.1 Definition of the total toxicity profile

We assessed toxicity for each system and graded it using a standard reference, such as the common terminology criteria for adverse events (common toxicity criteria of the National Cancer Institute) 13. Multiple toxicity observations are available for each patient, but for the purpose of defining the DLT, we take the dichotomized approach: most dose-finding protocols define as DLT the occurrence of grades 3 and 4 nonhematological and grade 4 hematological toxicities.

The same grade assessed on two different organs can correspond to a very different clinical importance, for example, a grade 3 nephrotoxicity and a grade 3 fatigue. As suggested by Bekele and Thall 4, we define weights reflecting the relative clinical importance of each grade and type of toxicity, rather than direct utilization of raw common terminology criteria for adverse events grades. Let *W* = {*w*_*l*,*j*_}be the matrix of weights defined for each grade *j*, *j* ∈ {0, … ,4}, of each toxicity type *l*, *l* ∈ {1, … ,*L*}.


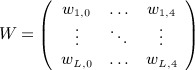


We realize the elicitation of the matrix of numerical weights in close collaboration with physicians before the initiation of the trial. We limit the space of toxicity grades to the range [0,4] because the occurrence of a grade 5, corresponding to death, would require another kind of decision rule, with direct interaction between the safety committee and the physicians to interpret the event and make a decision concerning the continuation of the trial. The matrix will be filled by values equal to 0 for the grades that do not exist in the grading system (e.g., headache grade 4).

To capture the relative clinical importance of various toxicity profiles (TPs) in this multidimensional space, we have proposed a flexible toxicity endpoint, thereafter called TTP, as an alternative measure to the TTB proposed by Bekele and Thall 4. As the same arithmetic sum of toxicity, defining the TTB, can correspond to clinical situations of different toxicity burdens, we computed the TTP as the Euclidean norm of the weights, which measures the length of the toxicity vector in a multidimensional space.

For patient *i* treated at dose level *d*_*k*_, the Euclidean norm *TTP*_*i*,*k*_ is defined by


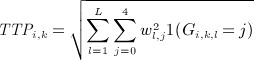
(1)

with, for patient *i* treated at dose *d*_*k*_,


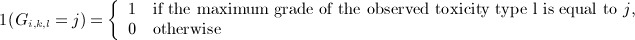


As there are a limited number of combinations of weights, the resulting score is, by construction, a quasi-continuous variable.

#### 2.1.2 Elicitation of the target total toxicity profile, *θ*

The use of this new toxicity endpoint leads us to define the target TTP, *θ*, that is, a TTP value judged acceptable by the clinicians. We interviewed expert clinicians to decide, for a set of various hypothetical cohorts, whether to escalate, repeat, or de-escalate the dose for the next cohort. Once a consistent classification of the cohorts is obtained, defined as a string of decisions of escalation corresponding to the lower values of TTP in the cohorts, followed by the decisions of repeat and the decisions of de-escalation corresponding to the higher TTP, we then compute the target TTP *θ* as the mean of TTPs of the cohorts associated with a decision to repeat the dose.

#### 2.1.3. Normalization

To work with a toxicity measure ranging from 0 to 1 that can be modeled using a quasi-Bernoulli likelihood 14, we subsequently normalize the TTP (nTTP) as


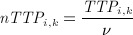
(2)

where *ν* is a normalization constant, *ν* = *TTP*_max_ + *ϵ*, with *TTP*_max_ equal to the most severe possible TP that we can compute from the matrix of the selected toxicities, and *ϵ* a small positive value. As the matrix of weights must be *a priori* defined and consequently includes a selection of expected toxicity items, *ϵ* thus allows us to adaptively integrate a severe toxicity that would not have been *a priori* selected and weighted during the trial.

We similarly normalize the target TTP, *θ*, to obtain *θ*^ * ^ defined as


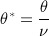


### 2.2. The quasi-likelihood continual reassessment method

The QLCRM that we propose combines the CRM using a logistic model in the frequentist framework 10 with the quasi-Bernoulli likelihood 14, allowing us to model the relationship between dose level and the quasi-continuous endpoint, nTTP.

#### 2.2.1. Model

Because *nTTP* ∈ [0,1], we can see it as a fractional event, and we can model the dose–toxicity relationship through a one-parameter logistic model as


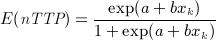
(3)

where *a* ∈  and *b* ∈  ^ + ^. We obtain the pseudo-doses *x*_*k*_ by the backward substitution that ensures that the dose–toxicity model (3) provides an exact fit over the initial guesses of toxicity scores, reflecting clinicians’ prior belief.

For our method, we fixed the intercept value *a*, leading to a one-parameter logistic model with only one parameter to be estimated, *b*, as recommended by Chevret *et al*. for the CRM with the binary endpoint 15.

#### 2.2.2. Variance specification

Considering the nTTP as a fractional event leads to the quasi-Bernoulli likelihood function in approximation 14–6, where a Bernoulli variance is embedded by default:


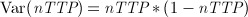


An alternative variance function is the Wedderburn variance 16,


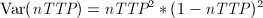


We may also explicitly derive the analytical variance function by considering the structure of nTTP score as a function of several toxicity random variables, using the delta method (equation given in the Appendix A).

As discussed later, a model based on this analytical variance function leads to an alternative approach that is outside the scope of the current research work.

#### 2.2.3. Likelihood

As performed by Yuan *et al*. for its toxicity score 6, we modeled the normalized nTTP using the quasi-Bernoulli likelihood that can accommodate fractional events. Assume that the last patient *i*, treated at dose level *d*_*k*_, corresponding to the pseudo-dose *x*_*k*_, experiences a toxicity measured by *nTTP*_*i*_. Its contribution to the quasi-Bernoulli likelihood will be


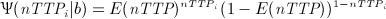
(4)

After the observation of the *i*th patient, we update the quasi-Bernoulli likelihood *L*_*i*_ by


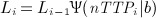
(5)

We also extended the quasi-likelihood function using the Wedderburn variance function 16.

#### 2.2.4. Decision rules

In the dose escalation phase of the trial, the dose allocated to the next cohort of patients is the dose associated with an estimated nTTP, 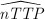, closest to the normalized target, *θ*^ * ^. At the end of the trial, we define the RD as the dose that would be allocated to the next cohort, that is, the closest to *θ*^ * ^.

As we work in a frequentist framework, likelihood-based estimates are not available to us before any toxicity has been observed. For this reason, the QLCRM includes two different stages, with an escalation stage procedure 12 as long as no toxicity event is observed (all nTTPs equal 0). The second stage of the design, which includes a model-based estimation of the dose–toxicity relationship, starts as soon as some heterogeneity in the toxicity response is observed. One advantage of using the TTP score, rather than the binary endpoint, DLT, is that the estimation of the model is possible from the first observation of toxicity, even mild, shorting greatly the first stage of this design.

### 2.3. Alternative designs

#### 2.3.1. Quasi-continual reassessment method approach

Yuan *et al*. previously proposed a design, hereafter referred to as QCRM, combining the CRM with the quasi-Bernoulli likelihood 6. The QCRM differs from the QLCRM in that it uses a Bayesian framework and models the dose–toxicity relationship using an empiric model as


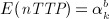


where *α*_*k*_ are defined by the working model with *α*_*k*_ ∈ [0,1] and *b* is the parameter to be estimated.

We can obtain the contribution to the model of the observation of the last patient by formulas (4) and (5).

Suppose that *g*_0_(*b*) is the prior distribution for the parameter *b*. After the observation of the *i*th patient, we update the quasi-posterior density for *b* by


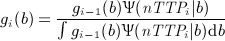


The decision rules are similar to that of the QLCRM.

#### 2.3.2. Extended isotonic design

The design proposed by Chen *et al*. is based on an isotonic regression for both the dose escalation phase and the identification of the RD 3.

Assume that the last patient *i* is treated at dose *d*_*k*_. We estimate the mean score, 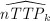, at each dose level. If a nondecreasing dose–toxicity relationship is observed, the score estimated by the isotonic regression, 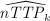, corresponds to the mean score (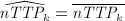). When it is violated, we use a pooled adjacent violator algorithm to estimate the scores. If the dose *k* + 1 has not been explored yet, its estimated score, 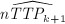, is equal to the score estimated at the highest dose explored *k* (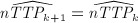).

We define the dose allocation rule, also used for the identification of the RD, as follows:

- If 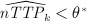, then if 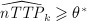, we assign the next patient to dose *d*_*k* + 1_, where *k* < *K*. Otherwise, we assign the next patient to dose *d*_*k*_.
- If 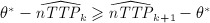, then if 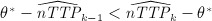, we assign the next patient to dose *d*_*k* − 1_, where *k* > 1. Otherwise, we assign the next patient to dose *d*_*k*_.

#### 2.3.3. Unified approach

The dose escalation algorithm is derived from the up-and-down method 17 and is based on a *t*-statistic 7.

Assume that the last patient *i* is treated at dose *d*_*k*_. Let *T*_*k*_ denote the *t*-statistic performed after the observation of patient *i*.


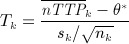


where 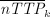 and *s*_*k*_ are the mean and the variance of nTTP computed from all available observations, *n*_*k*_, of the patients treated at dose *d*_*k*_, respectively.

We define the dose allocation algorithm as follows:

- If *T*_*k*_ *≤* − Δ, we assign the next subject to dose *d*_*k* + 1_.
- If *T*_*k*_ *≥*Δ, we assign the next subject to dose *d*_*k* − 1_.
- If − Δ < *T*_*k*_ < Δ, we assign the next subject to dose *d*_*k*_.

Δ is the design parameter that is fixed before the beginning of the trial; for further details, see 7. In contrast to the model-based methods previously described, which use information from all patients treated onto the trial until this time point, the dose allocation for the next cohort is based only on the observations of the patients treated at the last dose level.

At the end of the trial, we performed an isotonic regression using a pool adjacent violator algorithm if a nonmonotonic dose–toxicity relationship is observed. We then define the RD as the dose with an estimated nTTP, 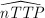, closest to the normalized target, *θ*^ * ^. After correction of the violators, it can happen that two or more doses are associated with the same estimated nTTP. Following the authors’ recommendations, if this estimated nTTP is the closest to *θ*^ * ^ and is above *θ*^ * ^, we select the lowest of these doses as the RD. If this estimated nTTP is the closest to *θ*^ * ^ but is below *θ*^ * ^, we select the highest of these doses as the RD.

### 2.4. Design parameters

To be consistent through the different methods, we only considered trials of fixed sample size. The trial stops when the prespecified number of patients, *n*, is exhausted. We did not allow skipping of dose levels during dose escalation.

We used the same definition of the RD in all the methods, that is, the dose associated with an estimated 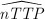 the closest to the normalized target *θ*^ * ^, even if above *θ*^ * ^, or a dose not allocated. Let us note that the UA cannot recommend a dose never explored as the isotonic regression is applied only to the doses already explored.

We performed a sensitivity analysis, for the QLCRM, QCRM, and EID designs, whereby the RD, although close to the normalized target *θ*^ * ^, should be among the doses allocated in the trial, as used in the design of Chen 3.

In the main analysis, the design parameters of QLCRM were as follows: we obtain the vector of initial guesses, also called working model, with the getprior function of the R-package dfcrm developed by Cheung 18, using dose 3 as the prior guess of RD, 0.04 as the indifference interval parameter, and a logistic model with the intercept set at 3. Please refer to the paper of Lee 19 for more details. For the QCRM, we similarly generated the working model from the getprior function (the prior guess of RD equal to dose 3 and the indifference interval parameter equal to 0.04 and the empiric model). The prior distribution of the empiric model parameter *b* is the exponential distribution with mean 1, as proposed by the authors 6.

We studied some other parameters that will be discussed later. For the UA method, we fix the design parameter Δ to 1 as recommended by the author.

## 3. Simulations

We used simulations to compare these four designs.

### 3.1. Definition of the matrix of weights and the target of toxicity

Assume that the main expected toxicities related to the treatment can be represented by three types of toxicities supposed to be independent: renal, neurological, and hematological toxicities. For the purpose of our simulation study, we elicited the matrix of weights from an expert clinician as follows:


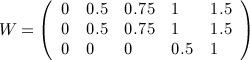


In this setting, we define DLT as the occurrence of a grade 3 or 4 neurological or renal toxicity or a grade 4 hematological toxicity.

With the use of this matrix, a patient with a DLT as single toxicity has a score equal to 1 for either a grade 3 renal, grade 3 neurological, or grade 4 hematological toxicity, and he has a score equal to 1.5 for a grade 4 renal or neurological toxicity. These patients experiencing a single DLT have almost the same score as a patient experiencing grade 2 renal plus grade 2 neurological toxicities. Lower weights give lower grades of toxicity.

The maximum TTP computed from this matrix is 2.34, corresponding to a grade 4 neurological toxicity, associated with grade 4 renal and grade 4 hematological toxicities. The TTPs were thus normalized by dividing each value by 2.5.

We asked the same expert to order a set of hypothetical cohorts of three patients with various TPs. The cohorts associated with the decision to repeat the dose have a mean nTTP varying from 0.24 to 0.32, corresponding for example to the following cohorts:

First cohort:

–Patient 1 presents grade 2 renal, grade 2 neurological, and grade 2 hematological toxicities. The nTTP of patient 1 is given by 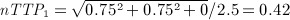.
–Patient 2 presents grade 1 renal, grade 1 neurological, and grade 3 hematological toxicities. The nTTP of patient 2 is given by 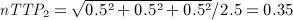.
–Patient 3 presents grade 0 renal, grade 0 neurological, and grade 0 hematological toxicities. The nTTP of patient 3 is given by 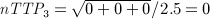.

The mean nTTP of this cohort, associated with no DLT, is


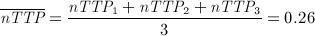


Second cohort, with different TPs:

- Patient 1 presents grade 3 renal (considered as DLT), grade 0 neurological, and grade 0 hematological toxicities. The nTTP of patient 1 is given by 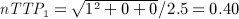.
- Patient 2 presents grade 0 renal, grade 2 neurological, and grade 1 hematological toxicities. The nTTP of patient 2 is given by 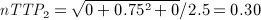.
- Patient 3 presents grade 1 renal, grade 0 neurological, and grade 2 hematological toxicities. The nTTP of the patient 3 is given by 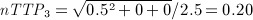.

The mean nTTP of the second cohort, associated with one DLT observed in patient 1, is


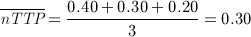


The target *θ*^ * ^ has consequently been set at 0.28.

### 3.2. Definition of the scenarios and generation of the toxicity data

We define each scenario by a given TP at each dose level. For each toxicity type, we defined the matrix of probabilities of observing grades 0 to 4, for the *K* dose levels. We assumed a unimodal distribution of the probability of observing a given grade across dose levels.

The probability of observing grade 0 (no toxicity) is maximal for *d*_1_ and decreases with the dose; the probability of observing a grade 4 increases with the dose, with a maximum probability at the last dose to be explored.

As these three toxicity types were assumed to be independent, we could compute for each dose level the probability of each of the 5^3^ combinations of the three toxicities (yielding a vector of 5^3^ probabilities corresponding to the 5^3^ TPs). For a given dose level, we then derive the mean nTTP and the probability of observing a DLT. The mean nTTP is the weighted sum of the 5^3^ TTP values. We detail one of the studied scenarios as an example in Appendix A.

Table 1 shows the description from the more toxic to the less toxic of the eight scenarios that we proposed. Scenarios C, F, and G represent a translation of scenario A to the right. We use these scenarios to study the impact of the position of the RD in the dose scale, for the same slope of the dose–toxicity curve around the RD. For all of them, the nTTP at the true RD is equal to the target nTTP. Scenarios B and E represent a mild variation of scenarios C and F, respectively, with an nTTP at the true RD slightly above the target nTTP. Similarly, scenarios D and H represent a mild variation of scenarios C and G, respectively, with an nTTP at the true RD slightly below the target nTTP.

**Table 1:** Description of the scenarios (mean nTTP and probability of DLT at each dose level).

	*d*_1_	*d*_2_	*d*_3_	*d*_4_	*d*_5_	*d*_6_
Scenario A
nTTP	0.183	**0.280**	*0.359*	0.409	0.432	0.439
nTTP-0.28	− 0.097	**0.000**	+*0.079*	+0.129	+0.152	+0.159
*p*(DLT)	0.195	**0.330**	*0.447*	0.512	0.557	0.558
Scenario B
nTTP	0.100	*0.198*	**0.310**	0.390	0.441	0.481
nTTP − 0.28	− 0.180	− *0.082*	+**0.029**	+0.110	+0.161	+0.201
*p*(DLT)	0.057	0.182	**0.379**	*0.491*	0.592	0.656
Scenario C
nTTP	0.108	0.183	**0.280**	*0.359*	0.409	0.432
nTTP − 0.28	− 0.172	− 0.097	**0.00**	+*0.079*	+0.129	+0.152
*p*(DLT)	0.065	0.195	**0.330**	*0.447*	0.512	0.557
Scenario D
nTTP	0.051	0.119	**0.270**	*0.370*	0.404	0.460
nTTP − 0.28	− 0.229	− 0.161	− **0.010**	+*0.090*	+0.124	+0.180
*p*(DLT)	0.002	0.014	**0.169**	*0.319*	0.417	0.554
Scenario E
nTTP	0.051	0.096	*0.188*	**0.312**	0.418	0.446
nTTP − 0.28	− 0.229	− 0.184	− *0.092*	+**0.032**	+0.138	+0.166
*p*(DLT)	0.002	0.014	*0.186*	**0.320**	0.506	0.554
Scenario F
nTTP	0.054	0.108	0.183	**0.280**	*0.359*	0.409
nTTP − 0.28	− 0.226	− 0.172	− 0.097	**0.000**	+*0.079*	+0.129
*p*(DLT)	0.011	0.065	0.195	**0.330**	*0.447*	0.512
Scenario G
nTTP	0.045	0.054	0.108	0.183	**0.280**	*0.359*
nTTP − 0.28	− 0.235	− 0.226	− 0.172	− 0.097	**0.000**	+*0.079*
p(DLT)	0.008	0.011	0.065	0.195	**0.330**	*0.447*
Scenario H
nTTP	0.051	0.111	0.141	0.189	**0.253**	*0.352*
nTTP − 0.28	− 0.229	− 0.169	− 0.139	− 0.091	− **0.027**	+*0.072*
*p*(DLT)	0.001	0.006	0.015	0.032	**0.097**	*0.196*

Bold entries correspond to values at the target dose. Entries in italics correspond to the values at the next closest dose to the target.

DLT, dose-limiting toxicity; nTTP, normalized total toxicity profile.

### 3.3. Simulation of the trials and metrics of comparison

For each scenario, we simulated 5000 repetitions of a trial of a fixed sample, *n*, recruiting patients by cohorts of three and exploring six dose levels. The detailed analysis describes the results for *n* = 36, in terms of percentage of dose recommendation and allocated dose percentage. We also reported the distribution of the patient toxicity scores nTTP and the number of DLTs observed in each trial, reflecting the safety of the different designs during the trial. The dose corresponding to the dose below the true RD is thereafter called *RD* − 1, and the dose just below *RD* − 1 is *RD* − 2; the dose above the true RD is called *RD* + 1, and the dose above *RD* + 1 is *RD* + 2.

Although actual phase I clinical trials samples are rather small, it is important to check if the methods converge asymptotically with increasing sample size. If a new method were found to fail to converge asymptotically, its performance would be questionable for usual small sample sizes. We studied the convergence of the PCS of the RD for *n* varying from 15 to 99, using a cohort size of three patients. The study was performed using R v2.11.1 20.

## 4. Results

### 4.1. Main results for *n* = 36

The distribution of dose recommendation obtained with the QLCRM is very narrow around the RD. The PCS is very high, varying from approximatively 80% to 90% according to the scenario (Table 2). In all cases, more than 90% of the recommendations correspond to the RD or the next closest dose. The control of overdosing is excellent with 0% of recommendations at *RD* + 2 in all scenarios. The chance of underdosing is also very low, and we never recommend the *RD* − 2.

**Table 2:** Recommendation percentage and allocated dose percentage, using the compared methods, for scenarios A, B, C, D, E, F, G, and H for 36 patients.

	Recommendation percentage						Allocated dose percentage					
	*d*_1_	*d*_2_	*d*_3_	*d*_4_	*d*_5_	*d*_6_	*d*_1_	*d*_2_	*d*_3_	*d*_4_	*d*_5_	*d*_6_
Scenario A	
QLCRM	3.4	**85.9**	*10.7*	0.0	0.0	0.0	19.4	**62.6**	*17.4*	0.6	0.0	0.0
QCRM	2.3	**83.6**	*14.1*	0.0	0.0	0.0	14.2	**63.1**	*22.0*	0.7	0.0	0.0
EID	11.3	**73.4**	*15.0*	0.3	0.0	0.0	21.5	**58.7**	*18.1*	1.7	0.1	0.0
UA	4.4	**86.4**	*9.1*	0.1	0.0	0.0	29.3	**59.2**	*11.3*	0.3	0.0	0.0
Scenario B	
QLCRM	0.0	*12.7*	**85.3**	2.0	0.0	0.0	9.1	*21.1*	**60.8**	8.9	0.2	0.0
QCRM	0.0	*10.5*	**87.4**	2.0	0.0	0.0	8.5	*18.7*	**63.1**	9.6	0.1	0.0
EID	0.3	*26.0*	**69.2**	4.4	0.1	0.0	9.1	*30.4*	**51.5**	8.4	0.5	0.0
UA	0.0	*18.9*	**79.6**	1.5	0.0	0.0	10.8	*36.7*	**48.7**	3.8	0.1	0.0
Scenario C	
QLCRM	0.0	3.0	**83.8**	*13.3*	0.0	0.0	9.2	14.9	**57.0**	*18.3*	0.7	0.0
QCRM	0.0	2.3	**84.1**	*13.6*	0.0	0.0	8.5	13.2	**58.5**	*19.3*	0.4	0.0
EID	0.3	12.6	**71.3**	*15.5*	0.4	0.0	9.1	21.2	**50.9**	*17.1*	1.7	0.1
UA	0.0	6.2	**84.4**	*9.4*	0.1	0.0	10.8	27.5	**51.5**	*10.0*	0.3	0.0
Scenario D	
QLCRM	0.0	0.1	**83.0**	*17.0*	0.0	0.0	8.4	8.6	**51.4**	*30.3*	1.4	0.0
QCRM	0.0	0.0	**82.5**	*17.5*	0.0	0.0	8.3	8.5	**50.2**	*32.1*	1.0	0.0
EID	0.0	0.9	**84.6**	*14.2*	0.2	0.0	8.3	10.2	**62.8**	*17.5*	1.1	0.0
UA	0.0	0.0	**93.4**	*6.5*	0.0	0.0	8.5	17.2	**61.0**	*13.2*	0.1	0.0
Scenario E	
QLCRM	0.0	0.0	*8.8*	**90.5**	0.6	0.0	8.4	8.4	*15.0*	**60.4**	7.8	0.1
QCRM	0.0	0.0	*8.2*	**91.4**	0.4	0.0	8.3	8.4	*14.6*	**62.4**	6.4	0.0
EID	0.0	0.1	*23.3*	**74.7**	1.8	0.1	8.3	8.6	*26.9*	**50.4**	5.6	0.2
UA	0.0	0.0	*16.0*	**83.6**	0.4	0.0	8.4	9.7	*34.4*	**45.0**	2.5	0.0
Scenario F	
QLCRM	0.0	0.0	2.7	**80.7**	*16.5*	0.0	8.4	8.5	13.1	**50.9**	*18.5*	0.6
QCRM	0.0	0.0	2.6	**84.7**	*12.7*	0.0	8.3	8.4	12.9	**54.9**	*15.3*	0.2
EID	0.0	0.4	13.4	**69.8**	*16.0*	0.5	8.3	9.2	20.6	**45.8**	*14.6*	1.6
UA	0.0	0.0	8.1	**81.4**	*10.4*	0.1	8.6	11.0	26.9	**44.9**	*8.5*	0.2
Scenario G	
QLCRM	0.0	0.0	0.0	2.6	**79.6**	*17.8*	8.4	8.3	8.4	12.3	**45.0**	*17.6*
QCRM	0.0	0.0	0.0	3.8	**85.6**	*10.6*	8.3	8.3	8.4	13.9	**50.8**	*10.2*
EID	0.0	0.0	0.3	13.3	**70.5**	*15.8*	8.3	8.4	9.1	19.0	**40.7**	*14.6*
UA	0.0	0.0	0.0	10.0	**79.1**	*10.9*	8.5	8.6	11.0	25.3	**39.3**	*7.4*
Scenario H	
QLCRM	0.0	0.0	0.0	1.8	**82.5**	*15.7*	8.3	8.4	8.7	15.0	**48.0**	*11.5*
QCRM	0.0	0.0	0.0	3.6	**88.7**	*7.7*	8.3	8.4	8.7	18.4	**50.9**	*5.3*
EID	0.0	0.0	0.1	4.9	**72.4**	*22.6*	8.3	8.4	8.8	13.7	**41.1**	*19.7*
UA	0.0	0.0	0.0	1.9	**78.2**	*19.9*	8.4	9.4	11.0	18.4	**39.5**	*13.3*

Results at the target dose are in bold. Results at the closest target dose are in italics.

QLCRM, quasi-likelihood continual reassessment method (our proposal); QCRM, quasi-continual reassessment method 6; EID, extended isotonic design 3; UA, unified algorithm 7.

With the QLCRM, more than 45% of the 36 patients are allocated at the true RD. The dose escalation process looks efficient with a percentage of patients allocated to the dose levels below the *RD* − 1 very close to 8.33% ( = 3/36) in all the scenarios where the true RD is above the *d*_2_, meaning that the dose is escalated after each cohort in most of the cases until the dose allocated is *RD* − 1.

The performance of the other methods is good in all the studied scenarios. The PCS varies from 82% to 91% in the QCRM, from 69 to 85% in the EID, and from 78% to 93% in the UA. The PCS of QLCRM and QCRM are very similar in the various scenarios, with a difference varying from − 6% to + 2%, the QCRM being slightly better than the QLCRM in six of the eight studied scenarios. The performance of the QLCRM is greater than that of the EID in all but one scenario, with a difference of PCS equal to or greater than + 9% in seven of the eight studied scenarios (up to + 16% in scenarios B and E). The difference of the performance of QLCRM compared with that of UA depends upon the scenario, varying from − 10% to + 7%. These two methods give similar results in four scenarios (A, C, F, and G), with a difference of PCS below 1%.

The distribution of allocated doses across the trial is very similar between QLCRM and QCRM, with more than half of the patients allocated to the RD in six of the eight scenarios. In all scenarios but one (scenario D), the parametric methods (QLCRM and QCRM) allocate more patients at the RD than the nonparametric methods (EID and UA). The distribution of allocated doses is wider with the EID; in particular, this method allocates more patients to *RD* + 2 in almost all the studied scenarios. The UA method appears more conservative in the dose escalation process, with much more patients allocated to the *RD* − 1 even when it is not the next closest dose to the target.

**Figure 1:**
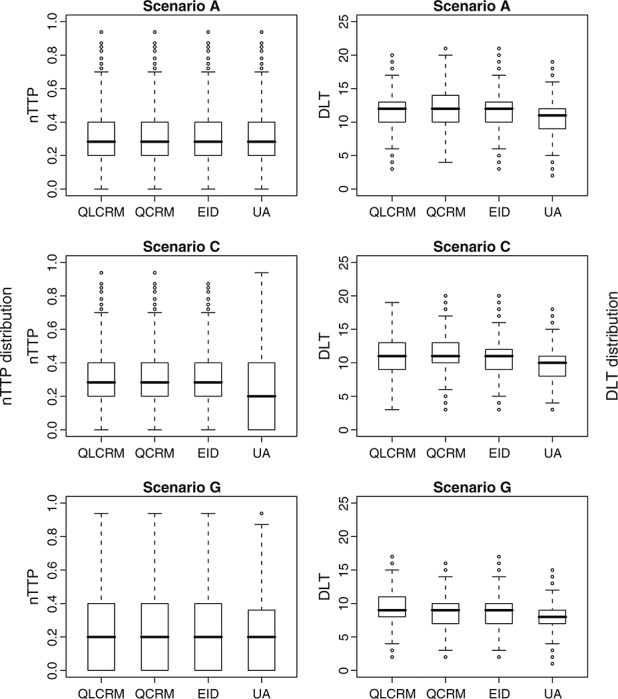
Box plot of normalized total toxicity profile (nTTP) and dose-limiting toxicity (DLT) distribution for the quasi-likelihood continual reassessment method (QLCRM), quasi-continual reassessment method (QCRM), extended isotonic design (EID) method, and unified approach (UA) method. We define the minimum whisker as *Q*_1_ − 1.5 * *IQR* and the maximum whisker as *Q*_3_ + 1.5 * *IQR*; *Q*_1_ and *Q*_3_ are the first and third quartiles, respectively.

The distributions of the observed nTTP scores and number of DLTs reflect the safety of the process for the patients included during the trial. These distributions are very similar for the different methods, across the eight studied scenarios, except for the UA method, which appears a little safer in terms of the number of DLT. Figure 1 illustrates the box plot of the nTTP and DLT distributions for scenarios A, C, and G (other scenarios are available on request).

When the Wedderburn variance function in QLCRM is used, the percentages of correct selection were similar numerically in most scenarios, but the PCS can be worse in some scenarios. In addition, we allocated more patients to the doses higher than the RD in all scenarios (details in Table 3 of Appendix A).

**A.1:** Recommendation percentage and allocated dose percentage, using Wedderburn and Bernoulli variances, for scenarios A, B, C, D, E, F, G, and H for 36 patients.

	Recommendation percentage						Allocated dose percentage					
	*d*_1_	*d*_2_	*d*_3_	*d*_4_	*d*_5_	*d*_6_	*d*_1_	*d*_2_	*d*_3_	*d*_4_	*d*_5_	*d*_6_
Scenario A	
QLCRMW	3.1	**83.4**	13.4	0.1	0.0	0.0	18.6	**60.5**	19.7	1.2	0.0	0.0
QLCRM	3.4	**85.9**	10.7	0.0	0.0	0.0	19.4	**62.6**	17.4	0.6	0.0	0.0
Scenario B	
QLCRMW	0.0	9.9	**85.5**	4.6	0.0	0.0	9.0	18.6	**58.6**	13.0	0.7	0.0
QLCRM	0.0	12.7	**85.3**	2.0	0.0	0.0	9.1	21.1	**60.8**	8.9	0.2	0.0
Scenario C	
QLCRMW	0.0	2.5	**77.2**	19.9	0.4	0.0	9.1	14.0	**52.5**	22.3	1.9	0.0
QLCRM	0.0	3.0	**83.8**	13.3	0.0	0.0	9.2	14.9	**57.0**	18.3	0.7	0.0
Scenario D	
QLCRMW	0.0	0.0	**65.0**	33.7	1.2	0.0	8.3	8.5	**38.4**	38.5	6.1	0.1
QLCRM	0.0	0.1	**83.0**	17.0	0.0	0.0	8.4	8.6	**51.4**	30.3	1.4	0.0
Scenario E	
QLCRMW	0.0	0.0	4.9	**88.7**	6.3	0.1	8.3	8.4	12.4	**53.5**	15.9	1.4
QLCRM	0.0	0.0	8.8	**90.5**	0.6	0.0	8.4	8.4	15.0	**60.4**	7.8	0.1
Scenario F	
QLCRMW	0.0	0.0	1.8	**67.9**	28.7	1.6	8.4	8.5	11.9	**43.7**	24.2	3.4
QLCRM	0.0	0.0	2.7	**80.7**	16.5	0.0	8.4	8.5	13.1	**50.9**	18.5	0.6
Scenario G	
QLCRMW	0.0	0.0	0.0	2.8	**61.8**	35.4	8.3	8.3	8.5	12.7	**36.7**	25.4
QLCRM	0.0	0.0	0.0	2.6	**79.6**	17.8	8.4	8.3	8.4	12.3	**45.0**	17.6
Scenario H	
QLCRMW	0.0	0.0	0.0	6.0	**73.2**	20.8	8.3	8.4	8.9	18.6	**41.6**	14.1
QLCRM	0.0	0.0	0.0	1.8	**82.5**	15.7	8.3	8.4	8.7	15.0	**48.0**	11.5

Results at the target dose are in bold.

QLCRMW, quasi-likelihood continual reassessment method using Wedderburn variance and its corresponding likelihood; QLCRM: quasi-likelihood continual reassessment method (our proposal, with the Bernoulli variance).

### 4.2. Convergence study

**Figure 2:**
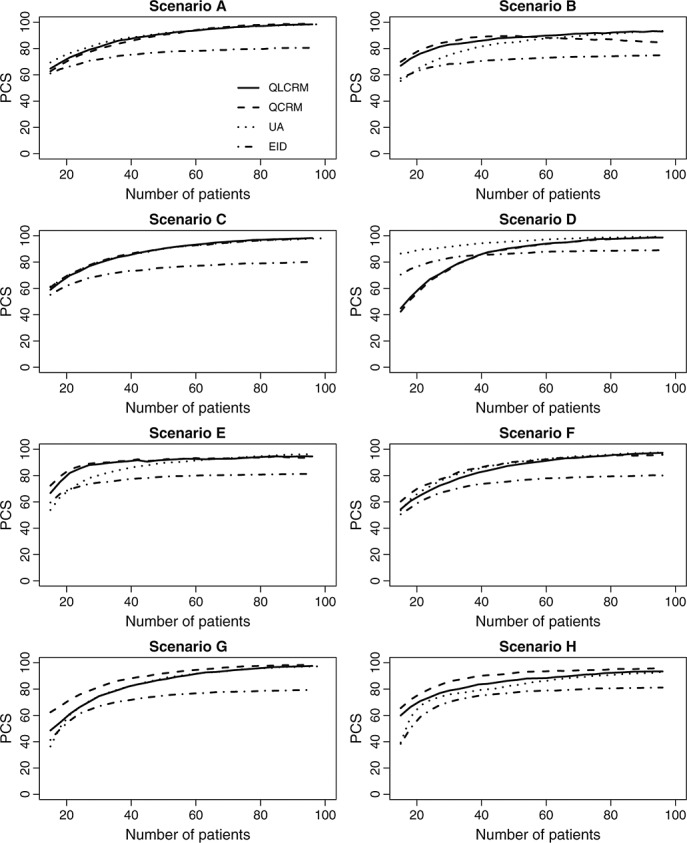
Convergence study for different methods: quasi-likelihood continual reassessment method (QLCRM), quasi-continual reassessment method (QCRM), extended isotonic design (EID), and unified approach (UA). PCS, percentage of correct selection.

The convergence study leads to the examination of the properties of the outlined designs for different numbers of patients. As illustrated on Figure 2, QLCRM, QCRM, and UA converge toward the true RD in all studied scenarios. For *n* = 99, the PCS is above 90% in all the scenarios. Contrasting with the excellent results observed with these three methods, the EID presents poor convergence behavior with a PCS plateauing very quickly as the number of patients increases, in six of the eight studied scenarios.

As phase I clinical trials generally include less than 50 patients, operating characteristics of the different methods for a small number of patients are also an important issue. QLCRM and QCRM show very similar properties, different from that of the UA design in three of the eight studied scenarios (the differences being in either directions). For these three designs, the PCS is greater than 65% when the sample size is equal to or greater than 24 in the eight studied scenarios.

### 4.3. Sensitivity analysis

To evaluate the impact of the decision rule of selecting the RD, we performed a sensitivity analysis for which the RD should be among the doses allocated in the trial, as recommended in the paper of Chen *et al*. 3. We did not observe any significant impact of the decision rule on the performance of the methods (details given in Appendix A).

## 5. Discussion

The emergence of molecularly targeted agents (MTA) in oncology revolutionizes the current phase I paradigm in a variety of ways 1–26. As noted by leaders in the field 25–26, the design of phase I trials is an open issue, and we require novel approaches to better fit with the particularities of MTA. One component of the current questioning concerns the choice of the best endpoint to identify the dose to be recommended for further evaluation. Although reasonable expectations come from the integration of alternate endpoints such as plasma drug concentration and dynamic biomarkers able to measure target inhibition in tumor or surrogate tissues 26–21, toxicity response remains a major endpoint in these trials 27–30. This was recently confirmed in a review of oncology phase I trials published between 1997 and 2008, including 99 trials evaluating MTA: in all study reports, the prespecified primary aim (or co-primary aim) was to determine the RD according to observed toxicity 31. An analysis of 82 phase I trials evaluating MTA as a single agent showed that DLTs were observed in only approximately half of the trials (43/82) 1.

To overcome these problems, several authors have recently proposed different toxicity scoring systems to measure quantitatively and comprehensively the overall severity of multiple toxicities per patient, considering a quasi-continuous toxicity endpoint 4,5. Bekele and Thall were the first to combine multiple graded toxicities in a single measure of toxicity endpoint (TTB) 4. In 2010, Lee *et al*. 5 proposed a toxicity burden score. Although they differed by the elicitation process, both scores are computed as an arithmetic sum of toxicity weights. By construction, multiple mild or moderate toxicities may easily lead to a higher score than a single DLT. In 2010, Chen *et al*. 3 defined an ETS preserving the relative order of the highest adjusted toxicity grade and its classification as a DLT or not, the additional toxicities counting only for a decimal part of the score. As underlined, these approaches lead to very different ranking of patients experiencing multiple moderate toxicities. Computed as the Euclidean norm, the nTTP is an intermediate alternative to the others. Its own mathematical property (triangular inequality) is appealing: the score of a single patient accumulating two toxicities is lower than the sum of scores of two different patients, each experiencing one toxicity event; in other words, multiple toxicities carry more weight if they come from different patients.

In this paper, we proposed a design based on a quasi-continuous toxicity score, the nTTP, and compared it with three other existing designs 6,7, using the same toxicity endpoint. A major aspect of our design is the construction of the matrix of weights and the definition of the toxicity target, which requires close collaboration with clinicians prior to the start of a trial. A panel of expert clinicians would have to ideally define the numerical weights and the target toxicity measure before launching a trial. The process elicitation needs a close collaboration with clinicians for each new phase I trial. Similar to how the definition of the DLT is tailored according to the context in a classical design, the weights allocated to each grade and type of toxicity could differ according to the trial population and the evaluated drug. The weights could also accommodate other features of the toxic event, such as its duration or reversibility. We acknowledge the fact that this design requires more collaborative efforts both at the design stage, for the elicitation of the matrix of clinical weights and to gauge the target score, and during the conduct of the study as the whole toxicity data have to be considered and collected in a timely fashion. Another hurdle is that the clinical meaning of the target score is less straightforward than a target percentage of DLT. The illustration of the target score by hypothetical cohorts of patients may help to facilitate its understanding. From the current results, we think that these issues are well balanced by the expected improvement in the performance of the dose-finding designs incorporating the nTTP endpoint.

Our simulation studies demonstrated that all these designs have good performance characteristics as it relates to the estimation of the (correct) RD. In particular, our design, the QLCRM, had a PCS of *≥*80% in all scenarios for a fixed sample size of 36 patients. For the model-based methods derived from the CRM, the choice of inference (frequentist versus Bayesian approach) and the type of one-parameter modeling (logistic versus empiric) have a limited impact on the performance, as illustrated by the very similar results of the QLCRM and the QCRM 6. The nonparametric methods utilized in the UA design 7 and the EID 3 utilized an isotonic regression-based approach for dose escalation. Whereas the PCS for the UA was similar to that of the model-based methods, the performance of the EID was less than optimal. The difference in the behavior of the evaluated methods in the convergence study was particularly striking: the EID design apparently did not converge to the true RD. This was likely due to recommending the upper dose in some cases when using the EID whereas model-based or UA designs would have recommended repeating the dose. This arises from the extrapolation of the estimated score at the highest allocated dose to the doses not explored yet in the case of EID. This is consistent with the conclusion of the comparison of several isotonic designs for dose finding using binary toxicity endpoint 32: the designs in which the dose closest to the RD is selected at every step, on the basis of isotonic regression 33, do not work well compared with the design where the isotonic regression is used only at the end of the trial on the cumulative cohort 34. On the other hand, the convergence study informed us on the good performance of the model-based methods as well as the UA design when the trial included 24 or more patients. All the designs yielded good overdosing control, with the UA design being more conservative by allocating more patients to doses lower than the RD.

In the main results, the QLCRM and QCRM used the working models obtained by the getprior function. We compared these results with those obtained with the working model published by Yuan *et al*. 6. For both QLCRM and QCRM, the choice of the working model between the two proposals has very little impact on the results except in scenario H where the performance is greatly impaired when using the working model of Yuan. We also compared the QLCRM, based on a one-parameter logistic model, for various values of the fixed intercept. The results are much better with the intercept value *a* = 3 than with other values (*a* = 2 or 5), which lead to incorrect identifying of the RD, whatever the working model we used: with *a* equal to 2, the RD is always overestimated, whereas with *a* equal to 5, the RD is underestimated (additional results available on request).

Considering a toxicity score as a fractional event and thus using a quasi-Bernoulli likelihood in the QLCRM modeling as performed by Yuan *et al*. 6 raised the issue of choice concerning variance function. We have studied this issue by comparing the results with the alternative Wedderburn variance. The design with the Wedderburn variance leads to similar performance in most scenarios but also results in poorer performance in some scenarios. We also derived the explicit variance function from the different toxicity components using the Delta method, leading to a more precise variance than Bernoulli variance or Wedderburn variance. As this variance function relies on different random variables, a joint modeling of the different toxicity components would be required, as performed by Bekele and Thall in their pioneering work for their toxicity score 4. However, the model would be more complex for a small potential additional benefit if we consider the rather good performance of the QLCRM. This could be the object of a future work as the original idea of our work was to propose an extension of the CRM based on the summarized measure of the different toxicities and not on multiple individual toxicity items.

In this work, we have proposed to extend the QCRM design proposed by Yuan *et al*. 6 to incorporate the TTP score in the QLCRM, using a logistic model in a frequentist framework, because we think that it is more intuitive for the clinicians. However, it is worth noting that we have extended the QCRM for three other variants: using a cloglog link function and an empiric modeling for the dose–toxicity relationship in a frequentist framework and using a logistic modeling in a Bayesian framework. The five variants present, on average, very similar results across the eight studied scenarios (results detailed in Table 4 of Appendix A). As proposed from the original CRM design for the DLT endpoint in 1996 10, the main purpose of these variants is not to make claims about which provides improvement over the other. Instead, the purpose is to add another perspective to the QCRM construction, to make it more general and to facilitate the properties of the design and its understanding. Because the performance of the proposed method is very similar with the different link functions, the choice of the underlying model may depend on the number of doses and the dose increment setup 11. A one-parameter logistic model may be more appropriate when a limited number of evenly spaced doses are explored, whereas an empiric function model would *a priori* fit better in case of a large number of dose levels with no relative or absolute dose increments. The choice of the inference is also a matter of debate despite its very limited impact on the performance of the method, as shown here when using a toxicity score, as well as by several authors for the classical DLT-driven CRM 10–11. The frequentist design requires two stages because the likelihood equation has no solution if no heterogeneity is observed in the response. An up-and-down escalation schema 12–17 is generally proposed for the first stage, as long as no toxicity event is observed. Even if the use of the classical schema in the beginning of a DLT-driven trial is reassuring for the clinicians, dose allocation may not be optimal until the model is fitted. One advantage of considering all grade information in the proposed design is that the model can be estimated from the first observation of toxicity, even if it is only a mild toxicity. This leads to a shorter first stage of the design and increases the percentage of patients allocated to the true RD (details available from the author upon request).

**A.2:** Recommendation percentage and allocated dose percentage, using the compared methods, for scenarios A, B, C, D, E, F, G, and H for 36 patients.

	Recommendation percentage						Allocated dose percentage					
	*d*_1_	*d*_2_	*d*_3_	*d*_4_	*d*_5_	*d*_6_	*d*_1_	*d*_2_	*d*_3_	*d*_4_	*d*_5_	*d*_6_
Allocated dose percentage	
QLCRM	3.4	**85.9**	*10.7*	0.0	0.0	0.0	19.4	**62.6**	*17.4*	0.6	0.0	0.0
QLCRMcl	3.4	**83.0**	*13.5*	0.0	0.0	0.0	18.6	**60.7**	*20.1*	0.6	0.0	0.0
QCRM	2.3	**83.6**	*14.1*	0.0	0.0	0.0	14.2	**63.1**	*22.0*	0.7	0.0	0.0
QCRM-EF	3.3	**86.2**	*10.5*	0.0	0.0	0.0	19.3	**62.7**	*17.4*	0.6	0.0	0.0
QCRM-LB	4.5	**86.3**	*9.2*	0.0	0.0	0.0	22.9	**62.3**	*14.3*	0.5	0.0	0.0
Scenario B	
QLCRM	0.0	*12.7*	**85.3**	2.0	0.0	0.0	9.1	*21.1*	**60.8**	8.9	0.2	0.0
QLCRMcl	0.0	*11.1*	**86.4**	2.5	0.0	0.0	9.0	*19.0*	**62.5**	9.5	0.0	0.0
QCRM	0.0	*10.5*	**87.4**	2.0	0.0	0.0	8.5	*18.7*	**63.1**	9.6	0.1	0.0
QCRM-EF	0.0	*12.6*	**85.5**	1.9	0.0	0.0	9.1	*21.0*	**61.1**	8.7	0.1	0.0
QCRM-LB	0.0	*15.5*	**83.3**	1.2	0.0	0.0	9.7	*23.5*	**59.3**	7.4	0.1	0.0
Scenario B	
QLCRM	0.0	3.0	**83.8**	*13.3*	0.0	0.0	9.2	14.9	**57.0**	*18.3*	0.7	0.0
QLCRMcl	0.0	2.5	**82.6**	*14.9*	0.0	0.0	9.1	13.8	**58.1**	*18.8*	0.2	0.0
QCRM	0.0	2.3	**84.1**	*13.6*	0.0	0.0	8.5	13.2	**58.5**	*19.3*	0.4	0.0
QCRM-EF	0.0	2.9	**83.8**	*13.3*	0.0	0.0	9.2	14.8	**57.2**	*18.2*	0.6	0.0
QCRM-LB	0.0	3.7	**85.7**	*10.6*	0.0	0.0	9.8	16.4	**57.5**	*15.7*	0.6	0.0
Scenario D	
QLCRM	0.0	0.1	**83.0**	*17.0*	0.0	0.0	8.4	8.6	**51.4**	*30.3*	1.4	0.0
QLCRMcl	0.0	0.1	**78.3**	*21.6*	0.0	0.0	8.3	8.5	**47.8**	*34.8*	0.5	0.0
QCRM	0.0	0.0	**82.5**	*17.5*	0.0	0.0	8.3	8.5	**50.2**	*32.1*	1.0	0.0
QCRM-EF	0.0	0.1	**82.4**	*17.5*	0.0	0.0	8.3	8.6	**51.0**	*30.7*	1.4	0.0
QCRM-LB	0.0	0.2	**86.9**	*12.9*	0.0	0.0	8.4	8.9	**55.1**	*26.4*	1.2	0.0
Scenario E	
QLCRM	0.0	0.0	*8.8*	**90.5**	0.6	0.0	8.4	8.4	*15.0*	**60.4**	7.8	0.1
QLCRMcl	0.0	0.0	*6.2*	**93.3**	0.5	0.0	8.3	8.4	*14.3*	**64.5**	4.5	0.0
QCRM	0.0	0.0	*8.2*	**91.4**	0.4	0.0	8.3	8.4	*14.6*	**62.4**	6.4	0.0
QCRM-EF	0.0	0.0	*8.5*	**90.8**	0.7	0.0	8.3	8.4	*15.0*	**60.7**	7.6	0.1
QCRM-LB	0.0	0.0	*11.1*	**88.5**	0.4	0.0	8.4	8.4	*16.8*	**59.3**	7.0	0.1
Scenario F	
QLCRM	0.0	0.0	2.7	**80.7**	*16.5*	0.0	8.4	8.5	13.1	**50.9**	*18.5*	0.6
QLCRMcl	0.0	0.0	2.4	**86.5**	*11.1*	0.0	8.4	8.5	13.4	**58.8**	*10.9*	0.0
QCRM	0.0	0.0	2.6	**84.7**	*12.7*	0.0	8.3	8.4	12.9	**54.9**	*15.3*	0.2
QCRM-EF	0.0	0.0	2.7	**81.4**	*15.9*	0.0	8.4	8.5	13.2	**51.8**	*17.7*	0.5
QCRM-LB	0.0	0.0	3.5	**83.8**	*12.7*	0.0	8.4	8.6	14.1	**51.9**	*16.4*	0.6
Scenario G	
QLCRM	0.0	0.0	0.0	2.6	**79.6**	*17.8*	8.4	8.3	8.4	12.3	**45.0**	*17.6*
QLCRMcl	0.0	0.0	0.0	6.8	**90.6**	*2.6*	8.3	8.3	8.5	18.8	**54.1**	*1.9*
QCRM	0.0	0.0	0.0	3.8	**85.6**	*10.6*	8.3	8.3	8.4	13.9	**50.8**	*10.2*
QCRM-EF	0.0	0.0	0.0	2.9	**80.3**	*16.8*	8.3	8.3	8.4	12.9	**46.1**	*15.9*
QCRM-LB	0.0	0.0	0.0	3.6	**81.3**	*15.1*	8.4	8.3	8.4	13.0	**45.8**	*16.1*
Scenario H	
QLCRM	0.0	0.0	0.0	1.8	**82.5**	*15.7*	8.3	8.4	8.7	15.0	**48.0**	*11.5*
QLCRMcl	0.0	0.0	0.0	15.8	**83.5**	*0.7*	8.3	8.4	9.1	30.8	**43.0**	*0.4*
QCRM	0.0	0.0	0.0	3.6	**88.7**	*7.7*	8.3	8.4	8.7	18.4	**50.9**	*5.3*
QCRM-EF	0.0	0.0	0.0	2.6	**84.1**	*13.3*	8.3	8.4	8.8	16.5	**48.7**	*9.3*
QCRM-LB	0.0	0.0	0.0	2.5	**85.3**	*12.2*	8.4	8.4	8.8	15.8	**48.6**	*10.1*

Results at the target dose are in bold. Results at the closest target dose are in italics.

QLCRM, quasi-likelihood continual reassessment method (our proposal: quasi-CRM with a logistic model in a frequentist framework); QLCRMcl, quasi-continual reassessment method with cloglog model in a frequentist framework; QCRM: quasi-continual reassessment method (6: quasi-CRM with an empiric model in a Bayesian framework); QCRM-EF, quasi-continual reassessment method with an empiric model in a frequentist framework; QCRM-LB, quasi-continual reassessment method with a logistic model, with a fixed intercept equal to 3, in a Bayesian framework assuming a normal law for the prior distribution of the slope, with mean equal to 0 and variance equal to 1.34.

Our design can be extended to do the following: (i) accommodate other quasi-continuous toxicity endpoints, such as those proposed by Bekele and Thall or by Chen *et al*. 4–3; (ii) adaptively integrate and update the toxicity matrix after the trial is launched to incorporate a new toxicity not selected *a priori* and weighted; (iii) integrate other stopping rules, as those proposed by Chen for the EID design 3 or by Zohar and Chevret for the CRM 35; and (iv) allow for different cohort sizes and/or skipping of dose levels during dose escalation.

## Appendix A

### A.1. Analytical variance function using the Delta method

We may explicitly derive the analytical variance function by considering the structure of nTTP score as a function of several toxicity random variables, using the delta method as


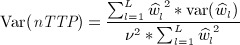


where 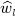 is the estimated weight of each toxicity type defined as


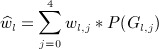


where *P*(*G*_*l*,*j*_) is the probability of grade *j* for toxicity *l* and


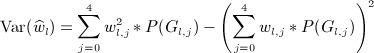


## A.2. Definition of the scenarios

In this section, we will detail how we generated the scenario F.

**Toxicity profile**

Let us define TP as the combination of a given grade *j*_r_ of renal toxicity, 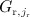, plus a given grade *j*_n_ of neurological toxicity, 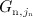, plus a given grade *j*_h_ of hematological toxicity, 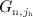, with *j*_r_, *j*_n_, and *j*_h_ being in {0,1,2,3,4}.

We can define a vector of the 5^3^ TPs.


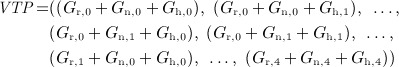


We compute the nTTP score of each toxicity profile, yielding a vector of 5^3^ nTTP values,


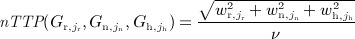


where 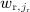, 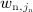, and 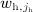 are the weights corresponding to the grade *j*_r_ of renal toxicity (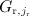), grade *j*_n_ of neurological toxicity (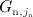), and grade *j*_h_ of hematological toxicity (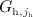), respectively, defined in weights matrix *W*, and *ν* is defined in Equation (2).

In the same way, we compute the DLT from the TP, leading to a vector of 5^3^ DLT values equal to 0 or 1.


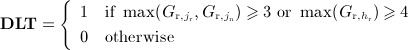


where *j*_r_, *j*_n_, and *j*_h_ are in {0,1,2,3,4}.

**Generation of the scenario**

We define a scenario by the probability of each of the 5^3^ TPs for the six dose levels. We can derive this from three matrices corresponding to each of the three toxicity types we consider. For each toxicity type, this matrix is a plausible matrix of the probabilities of observing each grade (from 0 to 4, defining the columns of the matrix) at each dose level (from *d*_1_ to *d*_6_, defining the rows of the matrix). Let *P*_r_, *P*_n_, and *P*_h_ be the probability matrices defined for the renal, neurological, and hematological toxicities, respectively. Find the matrices used for scenario F in the following.


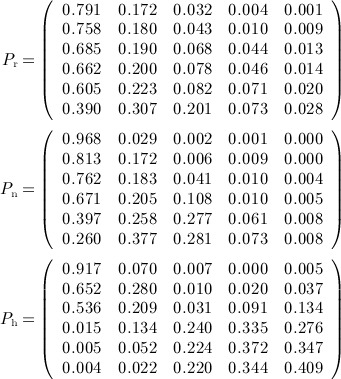


With the preceding three matrices, we can compute the probability of observing, at dose level *d*_*k*_, a given TP as


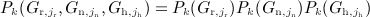


where *j*_r_, *j*_n_, and *j*_h_ are in {0,1,2,3,4}and 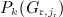 is the probability of observing a renal toxicity of grade *j*_r_ at dose level *d*_*k*_, defined in the matrix *P*_r_. We similarly define the probabilities 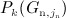 and 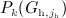 in the matrices *P*_n_ and *P*_h_, respectively.

For example, we compute the probability that a patient experiences grade 2 of renal toxicity, *G*_r,2_, plus grade 0 of neurological toxicity, *G*_n,0_, plus grade 1 of hematological toxicity, *G*_h,1_, at the dose level *d*_1_ as


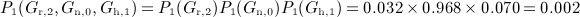


**Features of the scenario**

The mean of the normalized TTP at each dose level *d*_*k*_, 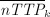, is the weighted sum of the 5^3^ nTTP values:


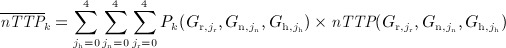


We can similarly compute the probability of DLT at each dose level *d*_*k*_ as


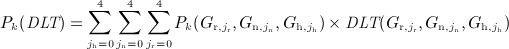


The probability matrices *P*_r_, *P*_n_, and *P*_h_ for all scenarios are available from authors on request.

## A.3. Sensitivity analysis

**Figure 3:**
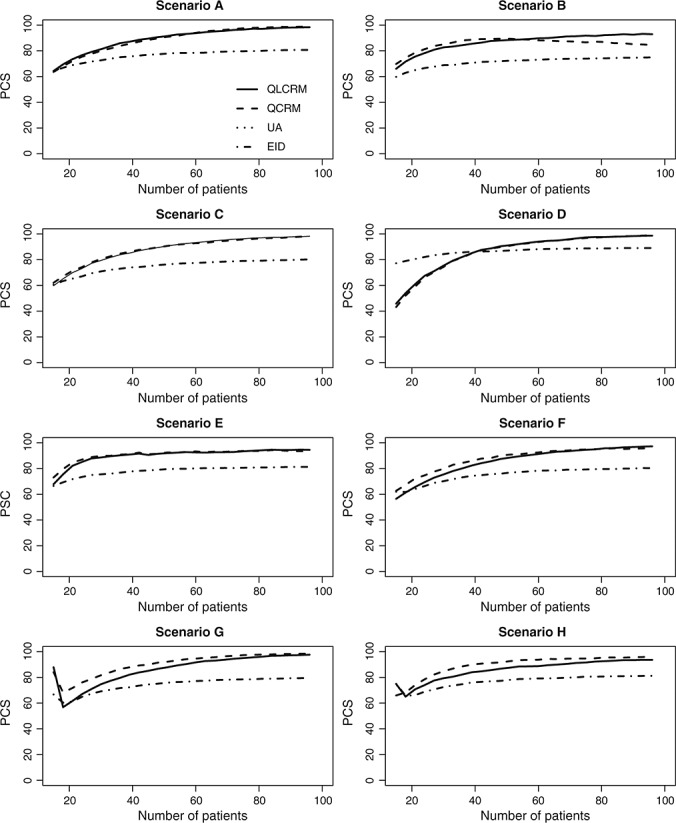
Sensitivity analysis for the convergence study for different methods: quasi-likelihood continual reassessment method (QLCRM), quasi-continual reassessment method (QCRM), extended isotonic design (EID), and unified approach (UA). PCS, percentage of correct selection.

As illustrated in Figure 3 of Appendix A, the results of the convergence rate were the same when the trial accrues more than 20 patients. In scenarios G and H, for which the true recommended dose is the fifth dose, the PCS is very high with the parametric methods for *n* = 15, that is, after having treated five cohorts.
